# Response to lorlatinib on a patient with *ALK*‐rearranged non‐small cell lung cancer harboring 1151Tins mutation with uterine metastasis

**DOI:** 10.1111/1759-7714.14056

**Published:** 2021-06-28

**Authors:** Takashi Kobayashi, Shintaro Kanda, Toshirou Fukushima, Takuro Noguchi, Nodoka Sekiguchi, Tomonobu Koizumi

**Affiliations:** ^1^ Department of Comprehensive Cancer Therapy Shinshu University School of Medicine Matsumoto Japan

**Keywords:** 1151Tins, ALK, gynecological metastasis, lorlatinib, non‐small cell lung cancer

## Abstract

We describe a case of an anaplastic lymphoma kinase (*ALK*)‐rearranged non‐small cell lung cancer with development of uterine metastasis after crizotinib and alectinib treatment. Gene analysis from the tissue of uterine metastasis revealed the presence of 1151Tins, which was considered to be a crizotinib and alectinib resistance mutation. Subsequent therapy with the third‐generation ALK inhibitor lorlatinib, but not ceritinib, showed antitumor activity for 1 year. The uterus is an uncommon site for metastasis from lung cancer, and our case indicated that serial gene analysis could provide new information about ALK inhibitor resistance.

## Graphical abstract

A patient with *ALK*‐rearranged advanced non‐small cell lung cancer presented acquired resistance to alectinib and uterine metastasis. Gene analysis using the tissue from the uterine metastasis revealed the presence of 1151Tins. Lorlatinib showed antitumor activity on this disease for one year.

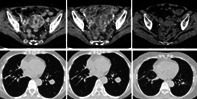

## INTRODUCTION

The discovery of rearrangement in the anaplastic lymphoma kinase (*ALK*) gene has led to a marked improvement in treatment strategy in patients with non‐small cell lung cancer (NSCLC). Several ALK inhibitors showed high response rates (60%–90%) and prolonged overall survival in patients with *ALK*‐rearranged NSCLC.^1^, ^2^, ^3^ On the other hand, resistant mechanisms to ALK‐inhibitors, such as secondary mutations in *ALK*, were analyzed in several studies^4^, ^5^, ^6^, ^7^, ^8^ and the clinical manifestations of unique metastatic organs in *ALK*‐rearranged lung cancer were also reported.^9^, ^10^, ^11^ We describe a case of *ALK*‐rearranged NSCLC who developed uterus metastasis during the treatment with two ALK inhibitors (crizotinib and alectinib). The diagnosis of uterus metastasis was confirmed by immunohistological analysis and determination of *ALK* fusion gene status with secondary mutations, 1151Tins, conferring resistance to crizotinib and alectinib. The new ALK inhibitor lorlatinib was useful to control the metastatic diseases. We report the clinical course and review of uterine metastases in NSCLC and secondary *ALK* mutation.

## CASE PRESENTATION

**FIGURE 1:**
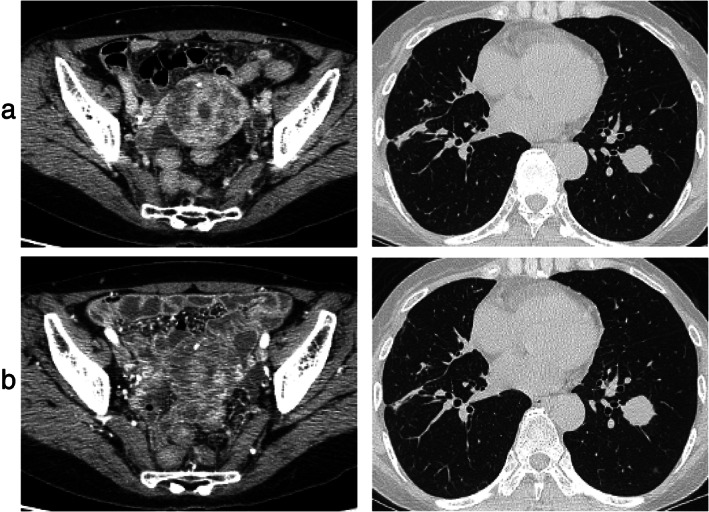
Lower abdominal and chest computed tomography findings at recurrence during alectinib therapy in the present case. (a) Uterus mass (arrows) and pulmonary mass (primary lesion in right lung and metastatic lesion in left lung). (b) Uterus and pulmonary mass after treatment with ceritinib in the present case

**FIGURE 2:**
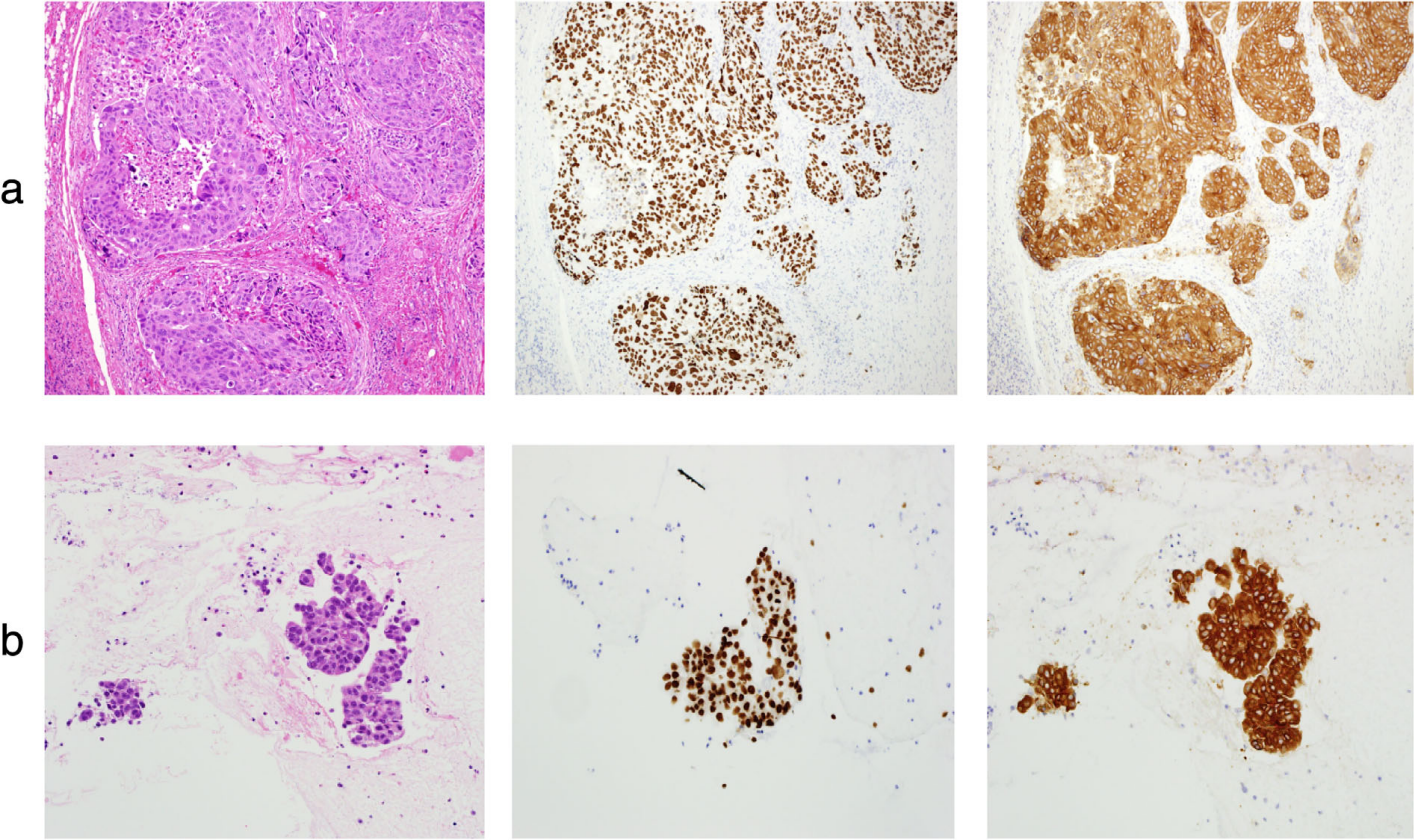
Pathological findings in cervical lymph node (a) and uterus (b). Left, hematoxylin and eosin staining; middle, immunohistochemical staining of thyroid transcription factor −1; right, immunohistochemical staining for anaplastic lymphoma kinase (ALK)

**FIGURE 3:**
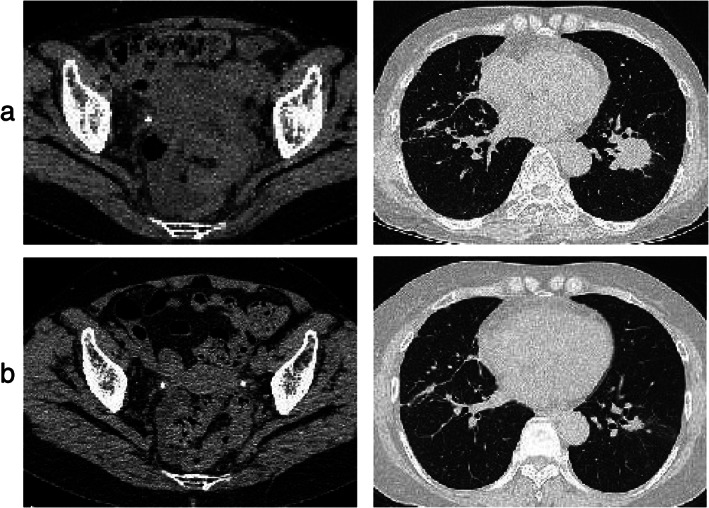
Lower abdominal and chest computed tomography findings before (a) and after (b) treatment with ceritinib in the present case

A 54‐year‐old woman was referred to our hospital about 10 years ago and diagnosed with stage IVA NSCLC [adenocarcinoma, T2bN3M1a (right pleura, left pulmonary), epidermal growth factor receptor (*EGFR*)‐wild type]. Seven years ago, re‐biopsy by bronchoscopy was performed due to progression after cytotoxic chemotherapies including cisplatin plus pemetrexed, docetaxel, vinorelbine, etc. Immunohistochemical analysis for ALK was positive and *ALK* fusion was detected by fluorescence in situ hybridization. The patient was therefore treated with crizotinib followed by alectinib. Partial response had been maintained for approximately 3 years, but she complained of lower abdominal pain. Abdominal computed tomography (CT) revealed a mass in the uterus (Figure 1a, left) and bilateral hydronephrosis. The uterus tumor caused postrenal acute renal failure due to bilateral ureter obstruction. Simultaneously, a palpable left cervical lymph node was observed, and chest CT also showed progression of primary and metastatic tumors (Figure 1a, right). A ureteral stent was implanted, which released the obstruction and resolved the renal failure. Cervical lymph node biopsy and endometrial curettage were performed. Histological findings confirmed both specimens to be metastases from the *ALK*‐rearranged NSCLC (Figure 2). In addition, gene analysis revealed the presence of 1151Tins in both specimens, which was considered to be a crizotinib and alectinib resistance mutation.^4^ She was treated with ceritinib. However, 3 months of ceritinib therapy failed to control the disease in both the uterus and pulmonary tumors (Figure 1b), and lower abdominal pain deteriorated. Subsequently, treatment was switched to lorlatinib because it became available for clinical use in Japan. She developed hyperlipidemia as an adverse event, but tumor reductions in both the uterus and pulmonary metastatic lesions were observed (Figure 3) and she has been treated for at least 1 year without recurrence. Analysis of *ALK* resistance mutations was performed by digital PCR using LBx Probe ALK (Riken Genesis, Tokyo, Japan) covering the *ALK* mutations 1151Tins, L1152R, C1156Y, I1171T, F1174L, V1180 L, L1196M, G1202R, S1206Y, G1269A, and L1198F.

## DISCUSSION

We present a case with *ALK*‐rearranged advanced NSCLC harboring uterine metastasis. Uterine metastasis occurs with low frequency during the clinical course of advanced NSCLC, so we searched such case reports in the PubMed database and summarize the case reports of NSCLC with uterine metastases and this case (Table 1).^12^, ^13^, ^14^, ^15^, ^16^, ^17^ Interestingly, several cases of *ALK*‐rearranged NSCLC presenting initially and/or developing ovarian and adnexa metastasis have also been reported.^10^, ^11^, ^12^, ^13^, ^14^, ^15^, ^16^, ^17^, ^18^, ^19^ These case reports and our case suggest that metastases to gynecological organs are not always rare in patients with *ALK*‐rearranged NSCLC. A retrospective study that investigated the metastatic patterns according to the molecular oncogene status reported that *ALK*‐rearranged NSCLC was associated with significantly increased numbers of metastatic sites in comparison to *EGFR*‐mutated and *ALK* wild‐type NSCLC.^8^ Thus, we should consider the possibility of gynecological metastasis in female patients with *ALK‐rearranged NSCLC*.

**TABLE 1:** Case reports of NSCLC with uterine metastases

Case	Age at uterus metastasis	Histology	Stage at initial diagnosis	Duration from initial diagnosis to uterus metastasis	Symptoms of uterus metastasis	Driver gene alteration	Ref.
1	73	Ad	IV	3 years	Vaginal bleeding	N/A	12
2	49	Ad	IV	Same time	No symptoms	N/A	13
3	69	Ad	IIIA	4 years	Vaginal bleeding	N/A	14
4	58	Ad	IV	10 months	Vaginal bleeding	N/A	15
5	55	Ad	IIIB	5 months	No symptoms	N/A	16
6	51	Ad	IV	Same time	No symptoms	EGFR	16
7	47	Ad	N/A	1 year 6 months	Vaginal bleedingAnemia	(−)	17
8	63	Ad	IIIB	2 years	Vaginal bleeding	EGFR	18
Current case	57	Ad	IV	3 years	Lower abdominal pain	ALK	‐

*Abbreviations*: Ad, adenocarcinoma; N/A, not evaluated.

1151Tins was reported as a secondary *ALK* mutation associated to crizotinib resistance at first,^4^ though it is rare among the resistance mutations reported to date.^5^ A preclinical study revealed that cells harboring 1151Tins with ALK fusion were resistant to crizotinib, ceritinib, and alectinib.^4^ Although we could not confirm the presence or absence of 1151Tins before crizotinib and alectinib treatment, it seemed that 1151Tins became dominant secondary during alectinib treatment as the acquired resistance mechanism because the tumor initially responded to crizotinib and alectinib. The other preclinical study reported lorlatinib activity for cells harboring 1151Tins with ALK rearrangement.^7^ In the present report, lorlatinib was effective for salvage therapy, while ceritinib was not. To our knowledge, this is the first case clinically proving the sensitivity of lorlatinib to *ALK*‐rearranged NSCLC harboring 1151Tins. Brigatinib is also a novel second‐generation ALK inhibitor, but its antitumor activity for cells harboring 1151Tins might be inferior to that of lorlatinib.^8^ Further information is needed regarding the optimal treatment sequence of these agents and mechanisms of resistance to each agent. To achieve longer survival in patients with *ALK* rearrangement, serial tissue samples should be taken by re‐biopsy and secondary *ALK* mutations conferring resistance to each ALK‐TKI should be evaluated.

In conclusion, we described an *ALK‐*rearranged NSCLC harboring 1151Tins mutation presenting a rare metastatic site of the gynecological tract successfully treated with loratinib. We emphasize that serial gene analysis could provide a better understanding of acquired resistance in *ALK‐*rearranged NSCLC and allow the selection of optimal treatment regimens.

## CONFLICT OF INTEREST

All the authors have no conflict of interest to declare.
